# Supplementary material for: Development and Comparison of Dengue Vulnerability Indices Using GIS-Based Multi-Criteria Decision Analysis in Lao PDR and Thailand
Source: Int J Environ Res Public Health. 2021 Sep 6;18(17):9421. doi: 10.3390/ijerph18179421 (PMC8430616; doi:10.3390/ijerph18179421)
Supplement: Supplementary file 1 [file ijerph-18-09421-s001.zip › ijerph-1348839-supplementary.pdf]

**Additional files**

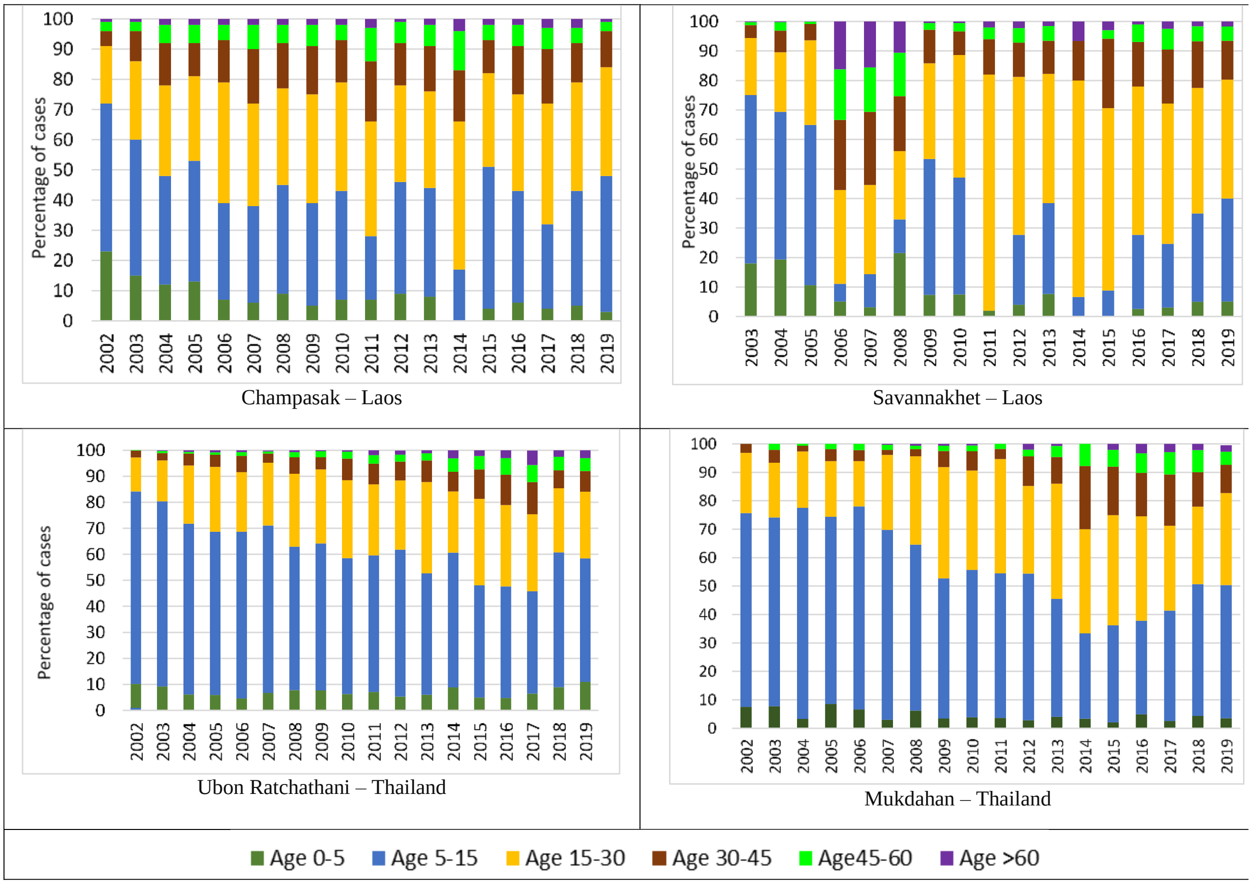


Figure S1. Age group distribution of reported dengue cases (DF, DHF, and DSS) in Savannakhet and Champasak provinces in Laos and Mukdahan and Ubon Ratchathani provinces in Thailand between 2002 and 2019.

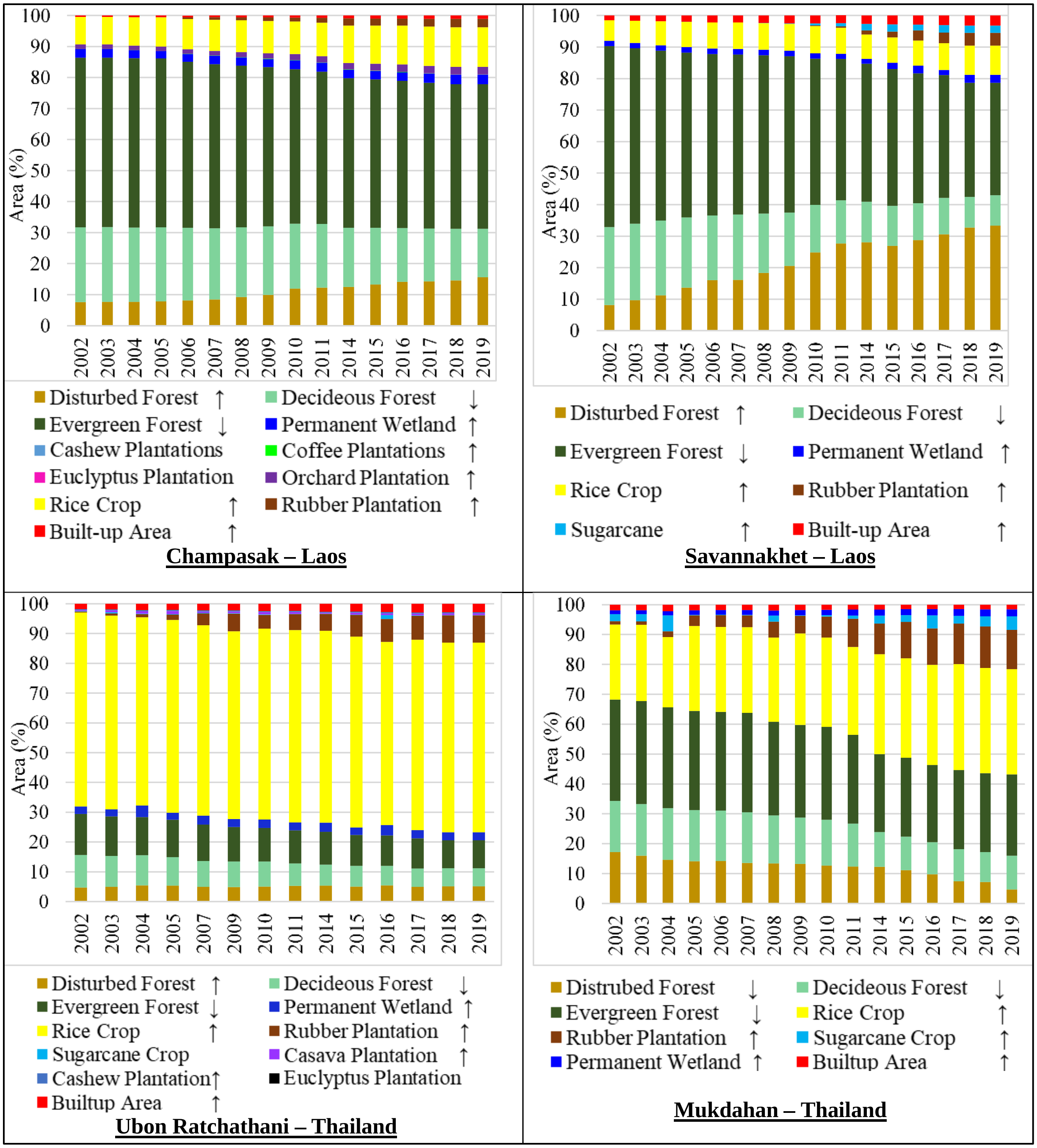


Figure S2. Landuse and landcover change in Champasak and Savannakhet provinces in Laos and Mukdahan and Ubon Ratchathani provinces in Thailand between 2002 and 2019.

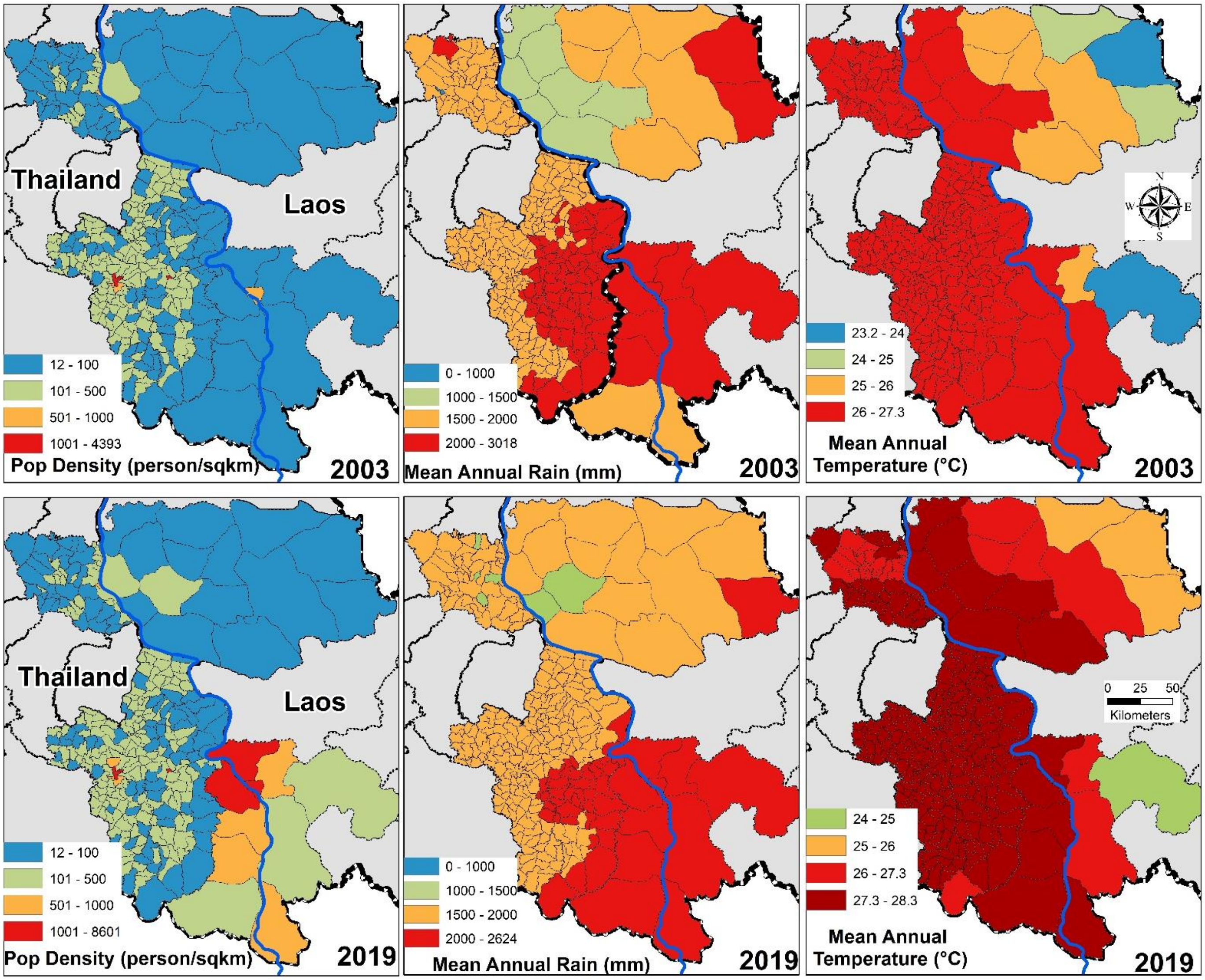


Figure S3: Change in three of the four exposure indicators; population density, mean annual rainfall, and mean annual temperature in selected provinces in Laos and Thailand between 2003 and 2019. The fourth exposure indicator, land environment change is shown in Figure 2 in the manuscript.

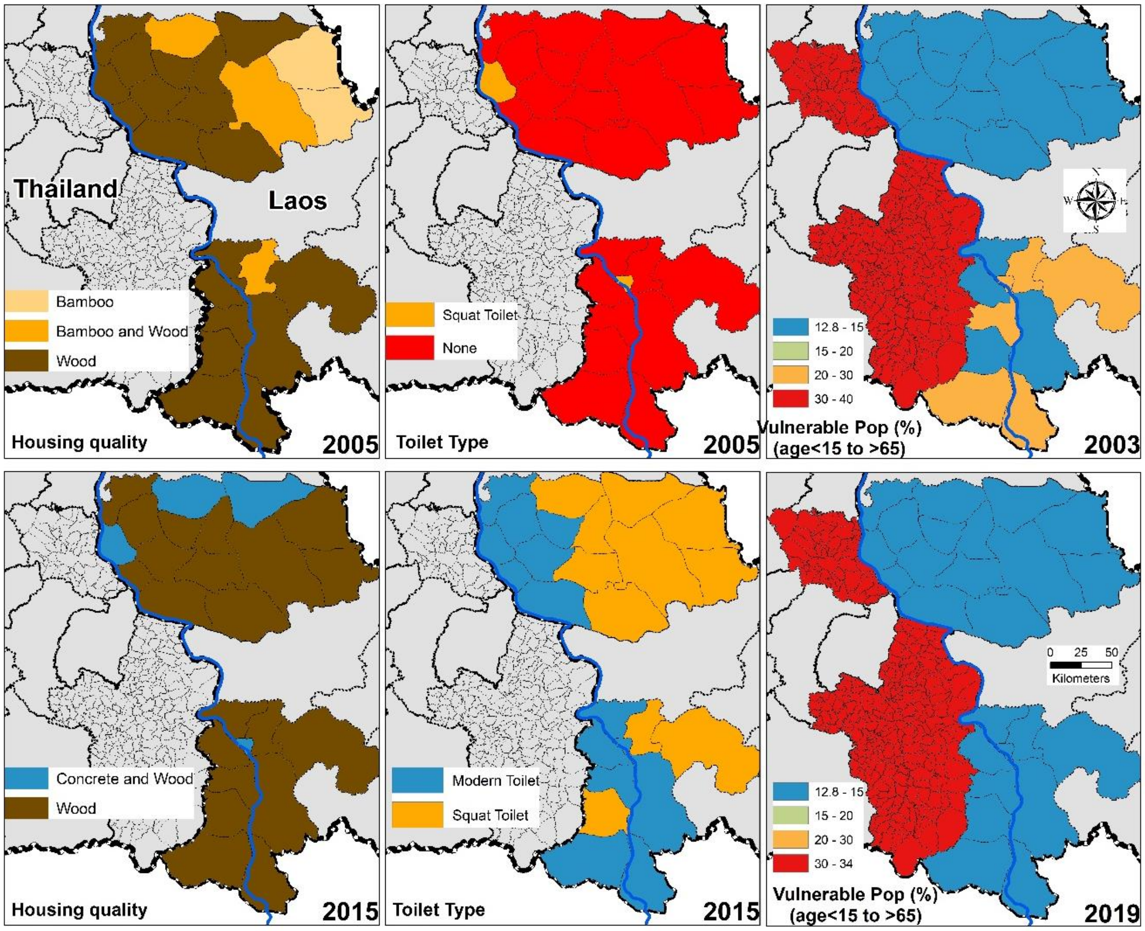


Figure S4: Change in the three susceptibility indicators, housing quality (left panels), toilet type (middle panels) and vulnerable populations (right panels) in Savannakhet and Champasak provinces in Laos in 2003 and in Mukdahan and Ubon Ratchathani provinces in Thailand in 2019 (Lao Statistics Bureau 2019; National Statistical Office of Thailand 2019).

- Housing quality for 2015 was assessed by categorizing districts into either having 25 - 50 % of village houses made of wood and concrete (blue) or 50 - 75% of village houses made wood (dark brown).
- Toilet type was assessed by categorizing districts into either having >50% of village houses with a squat toilet (yellow), >50% of village houses with a modern toilet (blue) or >50% of village houses with neither (red). Neither category likely consisted of open defecation.
- Vulnerable populations were assessed by % of the combined proportion of people <15 years and >60 years of age
- In Laos, 2005 census data were only available at the district level and here we presented these aggregated values.

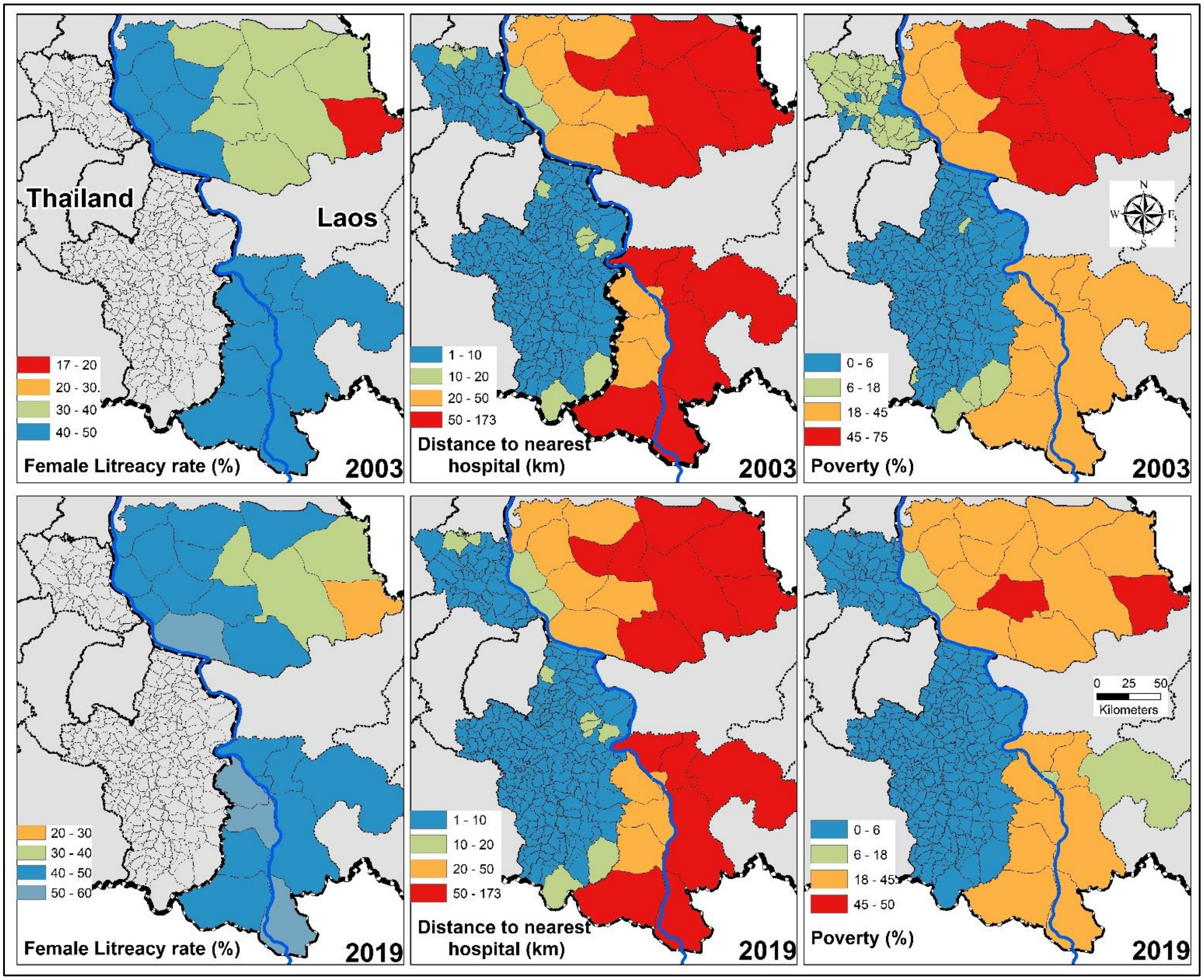


Figure S5: Change in indicators of adaptive capacity in selected provinces in Laos and Thailand between 2003 and 2019. Female literacy rate was not available for Thailand at subdistrict level. (Lao Statistics Bureau 2019; National Statistical Office of Thailand 2019).

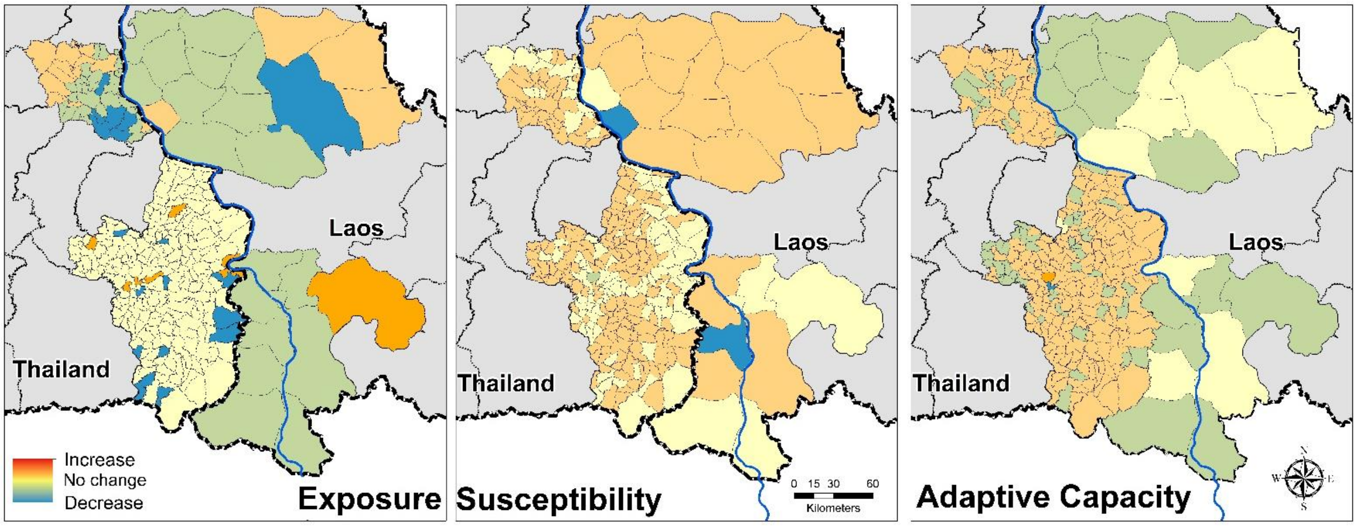


Figure S6: Change in exposure (left panel), susceptibility (middle panel) and adaptive capacity (right panel) based on the Water Associated Disease Index ($DVI_{WADI}$) in Savannakhet and Champasak provinces in Laos and Mukdahan and Ubon Ratchathani provinces in Thailand between 2003 and 2019.

Table S1: Indicator weight calculation using Shannon's Entropy (SE) for Laos

| **Determinants** | **Indicators (i)** | **Sub classes (j)** | **Observed dengue cases ($DP_i$)** | **Average population in subclass ij ($TP_i$)** | $d_{ij}$ | $P_{ij}$ | $\sum p_{ij}$ | $H_j$ | $1-H_j$ | $W_{ij}$ |
|---|---|---|---|---|---|---|---|---|---|---|
| Exposure | Cumulative rainfall in mm (1 month lag) | 0–300 | 48906 | 82978 | 0.59 | 0.91 | –0.31 | 0.12 | 0.88 | 0.08 |
| | | 300–1000 | 4806 | 81068 | 0.06 | 0.09 | | | | |
| | | >1000 | 70 | 144906 | 0.00 | 0.00 | | | | |
| | Mean temperature °C (1 month lag) | <20 | 880 | 60760 | 0.01 | 0.11 | –0.76 | 0.29 | 0.71 | 0.06 |
| | | >20 – <34 | 11325 | 122363 | 0.09 | 0.73 | | | | |
| | | >34 | 1490 | 77844 | 0.02 | 0.15 | | | | |
| | Forest area (%) | 0–10 | 0 | | | 0.00 | 0.00 | 0.00 | 1.00 | 0.09 |
| | | 10–60 | 33550 | 99515 | 0.34 | 0.52 | | | | |
| | | >60 | 20232 | 64742 | 0.31 | 0.48 | | | | |
| | Disturbed forest area (%) | 0–10 | 17927 | 67674 | 0.26 | 0.39 | –0.67 | 0.25 | 0.75 | 0.07 |
| | | 10–60 | 35855 | 86938 | 0.41 | 0.61 | | | | |
| | | >60 | 0 | 0 | 0 | 0.00 | | | | |
| | Plantation area (%) | 0–20 | 52433 | 80141 | 0.65 | 0.98 | 0.00 | 0.00 | 1.00 | 0.09 |
| | | 20–40 | 1349 | 133184 | 0.01 | 0.02 | | | | |
| | | >40 | 0 | | | 0.00 | | | | |
| | Rice and sugarcane crop area (%) | 0–20 | 34655 | 69565 | 0.50 | 0.74 | –0.59 | 0.22 | 0.78 | 0.07 |
| | | 20–40 | 18214 | 107949 | 0.17 | 0.25 | | | | |
| | | >40 | 913 | 333819 | 0.00 | 0.00 | | | | |
| | Built-up area (%) | 0–10 | 46204 | 78205 | 0.59 | 0.92 | –0.29 | 0.11 | 0.89 | 0.08 |
| | | 10–30 | 7578 | 142427 | 0.05 | 0.08 | | | | |
| | | >30 | | | | 0.00 | | | | |

| **Determinants** | **Indicators (i)** | **Sub classes (j)** | **Observed dengue cases ($DP_i$)** | **Average population in subclass ij ($TP_i$)** | **$d_{ij}$** | **$P_{ij}$** | **$\sum p_{ij}$** | **$H_j$** | **1–$H_j$** | **$W_{ij}$** |
|---|---|---|---|---|---|---|---|---|---|---|
| Exposure | Population density (person/sqkm) | 0–200 | 44890 | 66576 | 0.67 | 0.95 | –0.20 | 0.08 | 0.92 | 0.08 |
| | | 200–400 | 7656 | 227317 | 0.03 | 0.05 | | | | |
| | | >1000 | 1236 | 1135248 | 0.00 | 0.00 | | | | |
| | | Normal | 29568 | 62455 | 0.47 | 0.65 | | | | |
| | | none | 14979 | 60808 | 0.25 | 0.34 | | | | |
| Susceptibility | Age (%) | <15 and >60 | 32008 | 101692 | 0.31 | 0.54 | –0.69 | 0.26 | 0.74 | 0.06 |
| | | >15 and <60 | 18741 | 71160 | 0.26 | 0.46 | | | | |
| | Living conditions/ housing quality (%) | Houses made of concrete | 1404 | 107183 | 0.01 | 0.07 | –0.89 | 0.34 | 0.66 | 0.06 |
| | | Houses made of concrete and wood | 5400 | 80952 | 0.07 | 0.37 | | | | |
| | | Houses made of wood | 3888 | 39315 | 0.10 | 0.55 | | | | |
| | Toilet type (%) | Modern | 6202 | 919721 | 0.01 | 0.01 | –0.69 | 0.26 | 0.74 | 0.06 |
| | | Normal | 29568 | 62455 | 0.47 | 0.65 | | | | |
| | | none | 14979 | 60808 | 0.25 | 0.34 | | | | |
| Adaptive Capacity | Female literacy rate (%) | 0–50 | 51052 | 87570 | 0.58 | 0.90 | –0.32 | 0.12 | 0.88 | 0.08 |
| | | 50–100 | 2730 | 42792 | 0.06 | 0.10 | | | | |
| | Mean distance to hospital (km) | 0–10 | 0 | 0 | 0 | 0.00 | –0.69 | 0.26 | 0.74 | 0.06 |
| | | 10–20 | 26379 | 87068 | 0.30 | 0.47 | | | | |
| | | >20 | 27403 | 78920 | 0.35 | 0.53 | | | | |
| | Poverty incidence (%) | 0–20 | 7271 | 81208 | 0.09 | 0.14 | –0.76 | 0.29 | 0.71 | 0.06 |
| | | 20–40 | 39517 | 81208 | 0.49 | 0.73 | | | | |
| | | >40 | 6994 | 81209 | 0.09 | 0.13 | | | | |
| | | 50–100 | 2730 | 42792 | 0.06 | 0.10 | | | | |

Table S2: Indicators weight calculation using Shannon's Entropy (SE) for Thailand

| Determinants | Indicators (i) | Sub classes (j) | Observed dengue cases ($DP_i$) | Average population in subclass ($TP_i$) | $d_{ij}$ | $P_{ij}$ | ∑pij | Hi | 1–Hi | Wij |
|---|---|---|---|---|---|---|---|---|---|---|
| Exposure | Cumulative rainfall in mm (1 month lag) | 0–300 | 24398 | 7837 | 3.11 | 0.59 | | | | |
| | | 300–1000 | 17609 | 8233 | 2.14 | 0.41 | | | | |
| | | >1000 | | | | | –0.68 | 0.26 | 0.74 | 0.09 |
| | Mean temperature (°C) | 0 – 24 | 562 | 7367 | 0.08 | 0.01 | | | | |
| | | 24 – 28 | 21009 | 7904 | 2.66 | 0.51 | | | | |
| | | >28 | 20436 | 8156 | 2.51 | 0.48 | –0.76 | 0.29 | 0.71 | 0.09 |
| | Forest area (%) | 0–10 | 648 | 5853 | 0.11 | 0.12 | | | | |
| | | 10–60 | 3651 | 6562 | 0.56 | 0.58 | | | | |
| | | >60 | 1890 | 6422 | 0.29 | 0.31 | –0.93 | 0.35 | 0.65 | 0.08 |
| | Disturbed forest area (%) | 0–10 | 37990 | 435045900 | 0.00 | 0.25 | | | | |
| | | 10–60 | 3480 | 27171252 | 0.00 | 0.36 | | | | |
| | | >60 | 537 | 3943728 | 0.00 | 0.39 | –1.08 | 0.41 | 0.59 | 0.08 |
| | Plantation area (%) | 0–20 | 41447 | 7985 | 5.19 | 0.98 | | | | |
| | | 20–40 | 531 | 5355 | 0.10 | 0.02 | | | | |
| | | >40 | 29 | 3565 | | | –0.09 | 0.04 | 0.96 | 0.12 |
| | Rice and sugarcane crop area (%) | 0–20 | 12463 | 7948 | 1.57 | 0.30 | | | | |
| | | 20–40 | 10755 | 6799 | 1.58 | 0.30 | | | | |
| | | >40 | 18789 | 8805 | 2.13 | 0.40 | –1.09 | 0.41 | 0.59 | 0.07 |
| | Built-up area (%) | 0–10 | 40388 | 7669 | 5.27 | 0.99 | | | | |
| | | 10–30 | 1619 | 49551 | 0.03 | 0.01 | | | | |
| | | >30 | | | | | –0.04 | 0.01 | 0.99 | 0.13 |

| Determinants | Indicators (i) | Sub classes (j) | Observed dengue cases ($DP_i$) | Average population in subclass ($TP_i$) | $d_{ij}$ | $P_{ij}$ | ∑pij | Hi | 1–Hi | Wij |
|---|---|---|---|---|---|---|---|---|---|---|
| Exposure | Population density (person/sqkm) | 0–200 person per sqkm | 30768 | 6931 | 4.44 | 0.86 | | | | |
| | | 200–400 | 7750 | 11989 | 0.65 | 0.12 | | | | |
| | | >1000 | 3489 | 33900 | 0.10 | 0.02 | –0.47 | 0.18 | 0.82 | 0.10 |
| Susceptibility | Age (%) | <15 and >60 | 33167 | 12742 | 2.60 | 0.80 | | | | |
| | | >15 and <60 | 8833 | 13398 | 0.66 | 0.20 | –0.50 | 0.19 | 0.81 | 0.10 |
| Adaptive Capacity | Mean distance to hospital (km) | 0–10 | 30374 | 8158 | 3.72 | 0.70 | | | | |
| | | 10–20 | 9864 | 7239 | 1.36 | 0.26 | | | | |
| | | >20 | 1769 | 8948 | 0.20 | 0.04 | –0.72 | 0.27 | 0.73 | 0.09 |
| | Poverty incidence (%) | 0–20 | 41965 | 7937 | 5.29 | 1.00 | | | | |
| | | 20–40 | 4 | 5398 | 0.00 | 0.00 | | | | |
| | | >40 | 38 | 5173 | 0.01 | 0.00 | 0.00 | 0.00 | 1.00 | 0.13 |

Table S3: Pearson's correlation coefficients between each of three vulnerability indices and monthly dengue incidence per 100,000 persons per year in Laos and Thailand during 2003-2019. The $DVI_{BWM}$ for Thailand was not calculated because of lack of data at the subdistrict level. Coefficients in bold indicate significant correlations. $DVI_{WADI}$: Dengue Vulnerability Index – Water Associated Disease Index. $DVI_{BWM}$: Dengue Vulnerability Index - Best Worst Method. $DVI_{SE}$: Dengue Vulnerability Index - Shannon's Entropy.

| **Year** | **Laos** | | | **Thailand** | |
|---|---|---|---|---|---|
| | **$DVI_{WADI}$** | **$DVI_{BWM}$** | **$DVI_{SE}$** | **$DVI_{WADI}$** | **$DVI_{SE}$** |
| 2003 | 0.17 | 0.09 | **0.73** | **0.54** | 0.17 |
| 2004 | 0.44 | **0.75** | -0.34 | **0.81** | -0.25 |
| 2005 | 0.33 | 0.31 | 0.15 | **0.79** | 0.28 |
| 2006 | 0.37 | **0.80** | -0.39 | **0.86** | 0.40 |
| 2007 | 0.49 | 0.35 | -0.08 | **0.82** | -0.67 |
| 2008 | 0.44 | 0.47 | **0.75** | **0.92** | -0.34 |
| 2009 | 0.47 | 0.43 | **0.58** | **0.86** | -0.17 |
| 2010 | **0.53** | **0.54** | -0.27 | **0.90** | **0.79** |
| 2011 | **0.80** | **0.76** | -0.16 | **0.78** | -0.55 |
| 2012 | **0.57** | **0.59** | -0.40 | **0.67** | -0.22 |
| 2013 | 0.06 | 0.03 | **0.65** | **0.61** | **0.94** |
| 2014 | 0.03 | -0.02 | **0.56** | **0.82** | -0.38 |
| 2015 | -0.21 | -0.32 | 0.22 | **0.89** | 0.32 |
| 2016 | 0.10 | -0.08 | 0.11 | **0.77** | **0.71** |
| 2017 | **0.63** | **0.51** | 0.15 | **0.92** | 0.40 |
| 2018 | **0.64** | **0.55** | -0.39 | **0.86** | **0.72** |
| 2019 | 0.12 | 0.02 | **0.77** | 0.45 | 0.40 |

Table S4: Number of districts and percentage of with significant correlation coefficients (p-value < 0.05) and respective percentages out of the 25 districts of Savannakhet and Champasak in Laos and the 272 sub-districts of Mukdahan and Ubon Ratchathani in Thailand during 2003-2019. $DVI_{WADI}$: Dengue Vulnerability Index – Water Associated Disease Index. $DVI_{BWM}$: Dengue Vulnerability Index - Best Worst Method. $DVI_{SE}$: Dengue Vulnerability Index - Shannon's Entropy.

| | Laos | | | | | | Thailand | | | |
|---|---|---|---|---|---|---|---|---|---|---|
| Year | $DVI_{WADI}$ | | $DVI_{BWM}$ | | $DVI_{SE}$ | | $DVI_{WADI}$ | | $DVI_{SE}$ | |
| | Districts | | | | | | | | | |
| | No | % | No | % | No | % | No | % | No | % |
| 2003 | 4 | 16 | 3 | 12 | 6 | 24 | 59 | 22 | 53 | 19 |
| 2004 | 3 | 12 | 1 | 4 | 6 | 24 | 50 | 18 | 17 | 6 |
| 2005 | 4 | 16 | 3 | 12 | 8 | 32 | 46 | 17 | 23 | 8 |
| 2006 | 5 | 20 | 5 | 20 | 10 | 40 | 51 | 19 | 15 | 6 |
| 2007 | 5 | 20 | 5 | 20 | 7 | 28 | 79 | 29 | 23 | 8 |
| 2008 | 6 | 24 | 6 | 24 | 4 | 16 | 44 | 16 | 9 | 3 |
| 2009 | 2 | 8 | 1 | 4 | 5 | 20 | 50 | 18 | 13 | 5 |
| 2010 | 4 | 16 | 1 | 4 | 4 | 16 | 99 | 36 | 38 | 14 |
| 2011 | 10 | 40 | 5 | 20 | 6 | 24 | 61 | 22 | 25 | 9 |
| 2012 | 7 | 28 | 6 | 24 | 6 | 24 | 41 | 15 | 19 | 7 |
| 2013 | 2 | 8 | 1 | 4 | 5 | 20 | 94 | 35 | 96 | 35 |
| 2014 | 2 | 8 | 0 | 0 | 4 | 16 | 51 | 19 | 8 | 3 |
| 2015 | 0 | 0 | 0 | 0 | 6 | 24 | 144 | 53 | 45 | 17 |
| 2016 | 8 | 32 | 7 | 28 | 5 | 20 | 77 | 28 | 32 | 12 |
| 2017 | 9 | 36 | 4 | 16 | 3 | 12 | 80 | 29 | 33 | 12 |
| 2018 | 8 | 32 | 7 | 28 | 4 | 16 | 124 | 46 | 44 | 16 |
| 2019 | 1 | 4 | 1 | 4 | 5 | 20 | 87 | 32 | 103 | 38 |
| Average of the districts with >0.5 correlation | 5 | 19 | 3 | 14 | 6 | 22 | 73 | 27 | 35 | 13 |
| Outbreak year: Average of the districts with >0.5 correlation | 4 | 14 | 3 | 10 | 5 | 21 | 98 | 36 | 53 | 19 |

Table S5. The extent of inundated areas for annual flooding events in Savannakhet and Champasak provinces in Laos and Mukdahan and Ubon Ratchathani provinces in Thailand between 2002 and 2019. using Landsat (United States Geological Survey, 2020) and Sentinel-1 satellite images (European Space Agency, 2019). Overflowing of local rivers causes floods almost every year. Normalized difference water index (NDWI) was applied to extract the extent of water outside the riverbanks. The NDWI values range from -1 to +1. The NDWI values greater than 0 represent water/flooded areas (McFeeters, 1996). Flood extent from Sentinel-1 images was mapped using the focal median filter and applying a backscatter threshold value of -10 to -16  (Liu, 2016). A dash (-) represents no flooding in that particular year or province.

a. Laos

| **Year** | **Savannakhet** | | | **Champasak** | | |
|---|---|---|---|---|---|---|
| | **Start date** | **End date** | **Area Inundated (SqKm)** | **Start date** | **End date** | **Area Inundated (SqKm)** |
| 2002 | 10-Jul | 2-Aug | 1412 | 10-Jul | 2-Aug | 5862 |
| 2003 | - | - | - | - | - | - |
| 2004 | - | - | - | - | - | - |
| 2005 | 27-Jul | 19-Oct | 1145 | 27-Jul | 19-Oct | 3576.6 |
| 2006 | - | - | - | - | - | - |
| 2007 | 10-Oct | 30-Oct | 806 | - | - | - |
| 2008 | No satellite image | | | | | |
| 2009 | 10-Jul | 30-Oct | 4168 | 13-July | 14 October | 8794 |
| 2010 | 1-Oct | 7-Oct | 1006 | - | - | - |
| 2011 | 23-Jul | 1-Aug | 485 | 30-Jul | 27-Sep | 2643 |
| 2012 | - | - | - | - | - | - |
| 2013 | 7-Jul | 19-Jul | 404.5 | - | - | |
| 2014 | 27-Jul | 17-Sep | No satellite image | 27-Jul | 17-Sep | No satellite image |
| 2015 | 15-Jul | 30-Aug | 2187 | - | - | - |
| 2016 | - | - | - | 15 Jul | 24 Aug | 5722 |
| 2017 | 1-Jul | 30-Sep | 6489 | - | - | - |
| 2018 | 28-Jul | 27-Aug | 3400 | 28-Jul | 27-Aug | 3727.4 |
| 2019 | 31-Aug | 13-Oct | 2704 | 31-Aug | 1-Oct | 1960 |

b. Thailand

| Year | Ubon Ratchathani | | | Mukdahan | | |
|---|---|---|---|---|---|---|
| | **Start date** | **End date** | **Area Inundated (SqKm)** | **Start date** | **End date** | **Area Inundated (SqKm)** |
| 2002 | 10-Jul | 2-aug | 4104 | - | - | - |
| 2003 | - | - | - | - | - | - |
| 2004 | 8-Aug | 10 Oct | 3128 | - | - | - |
| 2005 | 27-Jul | 19-oct | 448.5 | - | - | - |
| 2006 | 3-Oct | 19-Nov | 1937 | - | - | - |
| 2007 | 10-Oct | 30-oct | 277 | - | - | - |
| 2008 | 5-Oct | 26-Nov | 473 | - | - | - |
| 2009 | 10-Jul | 30-oct | 1292 | - | - | - |
| 2010 | 1-Oct | 7-oct | 543 | - | - | - |
| 2011 | 23-Jul | 15-nov | 1024 | 23 -Jul | 1-Aug | 98.2 |
| 2012 | - | - | - | - | - | - |
| 2013 | 7-Jul | 19-jul | 643 | 23 - Sep | 29 Sep | 1.11 |
| 2014 | 27-Jul | 17-sep | 807 | - | - | - |
| 2015 | 15-Jul | 30-aug | 20 | - | - | - |
| 2016 | 01-Sep | 30-sep | 35 | - | - | - |
| 2017 | 1-Jul | 30-sep | 406 | 1-Jul | 30-Sep | 8.7 |
| 2018 | 25-Aug | 06-sep | 56 | 25-Aug | 06-Sep | 8.8 |
| 2019 | 31-Aug | 05-oct | 1309 | 31-Aug | 1-Oct | 54 |
